# Deep CNN for Indoor Localization in IoT-Sensor Systems

**DOI:** 10.3390/s19143127

**Published:** 2019-07-15

**Authors:** Wafa Njima, Iness Ahriz, Rafik Zayani, Michel Terre, Ridha Bouallegue

**Affiliations:** 1 Conservatoire National des Arts et Métiers, CEDRIC/ LAETITIA Laboratory, 75003 Paris, France; 2 University of Carthage, Higher School of Communication of Tunis, LR-11/TIC-03 Innov’COM Laboratory, 2083 Ariana, Tunisia

**Keywords:** Convolutional Neural Networks (CNN), deep learning, image classification, indoor localization, kurtosis, RSSI fingerprinting

## Abstract

Currently, indoor localization is among the most challenging issues related to the Internet of Things (IoT). Most of the state-of-the-art indoor localization solutions require a high computational complexity to achieve a satisfying localization accuracy and do not meet the memory limitations of IoT devices. In this paper, we develop a localization framework that shifts the online prediction complexity to an offline preprocessing step, based on Convolutional Neural Networks (CNN). Motivated by the outstanding performance of such networks in the image classification field, the indoor localization problem is formulated as 3D radio image-based region recognition. It aims to localize a sensor node accurately by determining its location region. 3D radio images are constructed based on Received Signal Strength Indicator (RSSI) fingerprints. The simulation results justify the choice of the different parameters, optimization algorithms, and model architectures used. Considering the trade-off between localization accuracy and computational complexity, our proposed method outperforms other popular approaches.

## 1. Introduction

The Internet of Things (IoT), also known as the Internet of Objects, is a trending concept intended as a network of interconnected smart objects receiving and sending data without human intervention [1,2,3]. The development of applications in IoT is strongly related to the notion of physical location and positions. Therefore, localization technologies will play an important role in the IoT and may become embedded into the infrastructure or into the object. In fact, collected data are reported for a specific IoT application, which requires dedicated data analytic tools to make sense of them and take the appropriate action. Plenty of applications are related to location-based services, which increases the importance of location information. This information can be used for target tracking, surveillance applications, guiding autonomous vehicles, etc. [4,5,6,7]. Therefore, collected data are meaningless if not combined with the accurate location of the concerned sensor node. The latter can be obtained by using Global Navigation Satellite Systems (GNSS), such as the Global Positioning System (GPS) [8], which is an efficient outdoor localization system. These solutions cannot be deployed in indoor environments due to the multipath effects caused by obstacles existing between satellites and users, which cause an important degradation of the signals. To overcome this limitation, the idea is to use radio signals for communication between objects. The communication technology is also a challenge in the IoT development, and the choice is closely related to the application. Bluetooth [9], Ultra-Wide Band (UWB) [10], Radio Frequency Identification (RFID) [11], and wireless local area network WiFi [12] have been widely used in indoor localization. Most proposed indoor localization systems are based on WiFi signals due to the wide use of mobile devices that support this technology. In fact, the other aforementioned communication solutions require specialized infrastructure (wireless radio beacons) to be installed in the indoor environment and extra equipment in the devices. Because the signal characteristics are strongly related to the distance between the transmitter and the receiver, they can be used to perform localization [13,14,15]. Usually, the easy to obtain parameters are the Received Signal Strength Indicator (RSSI) [16,17,18], Channel State Information (CSI) [19,20], Angle Of Arrival (AOA) [14,21], Time Of Arrival (TOA) [22], and time difference of arrival [23]. RSSI does not require specific hardware for time or phase synchronization, and no modification is needed on the device firmware to be able to acquire it. That is why it is the parameter being explored most today.

Based on RSSI signals, existing methods can be essentially classified into fingerprinting-based solutions and ranging-based solutions. The latter is combined with trilateration method and uses geometric properties to estimate the sensor’s location [24]. In such a solution, the distance to Reference Positions (RPs) is estimated by a propagation model, and at least four RPs are needed to get a 3D position. This requires that the RPs have known positions, which is not easy to obtain in real indoor scenes, this being the major drawback of the trilateration technique. Furthermore, the performance of such a technique depends on the number of RPs and the precision of the propagation model, which depends on the multipath effects. The fingerprinting method overcomes the mentioned drawbacks because it employs a constructed radio map to be compared to RSSI measurements associated with the sensor to localize [25]. It is a cost-effective solution, and its accuracy is related to the sufficiency of data, where huge RSSI databases are constructed and manipulated to achieve a good localization accuracy. This increases the complexity and the running time of fingerprint-based localization systems, making them not adapted to real-time localization and not able to deal with big sensor networks. Therefore, for a solution that uses a learned model, reducing the online complexity is extremely needed. Recently, promising indoor localization solutions were implemented based on RSSI fingerprinting combined with Machine Learning (ML) methods [26]. Since data preparation and preprocessing are assured in the offline/training phase, only the prediction task is performed online. Therefore, to find its position, a sensor node interrogates a trained model, which performs the estimated position. Such algorithms shift the computational complexity from the online/prediction process to the model offline/training step. Thus, such solutions based ML are highly recommended in real-time localization applications. This is motivated by the highly-efficient Deep Learning (DL) algorithms, which have been demonstrated to show very good performance in different contexts and applications related to the indoor localization field: LOS/NLOS identification [19,27], activity recognition [28], uncertainty prediction [29], denoising autoencoders [30], and localization [31,32]. These DL-based methods have been widely introduced into indoor localization, estimating either the location coordinates or other localization information such as room identification [31], floor identification [17], region identification [13,14,33], etc. Sound-based localization systems have been proposed in [13,14], ensuring a region identification prediction. The sensor data (sound) used require the use of specific hardware (microphones) to be measured. The authors in [33] detected the region of the sensor node and explored the nodes in the vicinity to estimate the sensor’s location based on RSSI measurements and geometric properties. The mentioned works were not flexible, due to the fact that they needed to place microphones or RPs at the top of each square region formed, which is impractical.

As said before, different localization approaches based on DL methods, like Support Vector Machine (SVM) [34] and Neural Networks (NN) [35], have been developed. Different types of NN have been used in the indoor localization context, especially Deep Neural Networks (DNN) and their variants: Multi-Layer Perceptron (MLP) [29], Recurrent Neural Networks (RNN) [16], Convolutional Neural Networks (CNN) [36,37], etc. The authors in [38] used a DNN to predict the node location coordinates (latitude and longitude) in a multi-building and multi-floor environment, achieving 9.29 m localization error. The approach implemented in [16] introduced RNN models as the DL method, where RSSI signals were used as input data and GPS coordinates were used as output neurons to train RNN models, in order to generate a model able to predict the location. The MLP introduced in [29] was applied to predict location uncertainty, while it was applied to RSSI statistics in [17] to predict the user’s floor. To deal with the constraints of network training and to reduce the number of neural network parameters (weights and biases) to learn and the complexity of traditional NN, Convolutional Neural Networks (CNN) [36] have been deployed. CNN is a class of deep NN that is widely used. It reduces the complexity of traditional NN and the number of weights to learn by its weight-sharing structure. This means that CNN requires less training parameters and can bring better generalization and robustness. Another reason why we use CNN is to deal with the need for large datasets required by traditional NN, to avoid overfitting problems. Another challenge in implementing positioning systems based on CNNs is that these networks have translational invariance. This feature coincides with the temporal dependency between RSSI fingerprints. Since this NN structure has high invariance in translation, it has been widely used in image processing and classification [39,40,41], achieving a spectacular success in this field. Thus, applying CNN on fingerprint images recently has arisen as an important interest in the localization community.

In [28], the researchers designed a CNN for a pedestrian activity recognition, which can serve as landmarks for indoor localization. Here, one-dimensional sensor data from accelerometers, magnetometers, gyroscopes, and barometers were considered as network inputs. This work needed specific types of sensors and did not take into consideration the energy consumption problem. In [13], the authors converted the sound signal collected by a microphone into a spectral map to input it into a CNN model. The authors in [19] used CNN to determine the NLOS channel classification and ranging error estimation based on UWB CIR data. Here, the CNN models used were fed one-dimensional input CIR images, then, to estimate the position, Least Square (LS) and Weighted Lest Square (WLS) algorithms were used, needing at least four detected access points (APs). Recently, other designed localization systems based on RSSI measurements were proposed. In [16], a hierarchical classifier employed a combination of smaller CNN models, which worked together to deliver a location prediction. This system took 2D RSSI images, where each image was of size (N×K), *N* was the number of training points, and *K* was the number of APs. The authors in [42] identified the location of a user (building ID and floor ID) by leveraging RSSI obtained from neighboring APs. From a given 1D RSSI fingerprint associated with a training point, a 2D image was made, adding some dummy values (for example: (23×23), 2D image was constructed from a (520×1) RSSI fingerprint where 520 is the number of APs, adding nine dummy data). A hierarchical CNN architecture was proposed in [18] using fingerprint images combining WiFi and magnetic field peculiarities in a single image. The WiFi branch and the magnetic one produced two different predictions. Then, the prediction vectors were combined as the input of a united branch to estimate the user’s location. A framework was implemented in [38] using CNN to determine the building ID and the floor ID, then a DNN was introduced to estimate the position’s coordinates. In this work, 2D RSSI images were considered where each image corresponded to a specific training point, i.e., an image was formed by RSSI measurements received by a training point from different APs at different instants.

Few existing localization solutions related to localization NN have explored 3D radio images. They have always been used when working on robots’ localization, due to the fact that multiple types of sensors are integrated on a robot (camera, laser, odometer, etc.) [43,44,45,46]. Therefore, each radio image plane contained data received from a specific sensor. Besides robot localization, 3D radio images can be used in systems exploring different types of data. For instance, the authors in [18] explored RSSI data accompanied by magnetic and acceleration information. Based on CSI, 3D radio images can be generated as developed in [20,47], where one CSI matrix of an antenna was considered as the red, green and blue planes of the image. Therefore, the image was constructed by combining three channels of CSI.

In this paper, we deal with the issue of indoor localization in the context of the IoT as a 3D radio image-fingerprint-based location recognition problem motivated by the outstanding performance of CNN in image classification problems and based on RSSI fingerprints. RSSI measurements can be significantly affected by noise and environmental changes. Different sources of RSSI measurement uncertainty were deeply analyzed in [48] in order to determine the impact of each disturbing phenomena on the localization accuracy. The authors in [49] evaluated the effect of different propagation conditions on the localization accuracy in order to predict a satisfying accuracy, performing a linearization process and a Kalman filter. However, this does not impact significantly the accuracy due to the correlated shadowing process. To minimize their temporal variation and fluctuation, during the 2017 IPIN competition explained in [50], the UMinho Team merged the fingerprints collected in the same position to generate a less noisy fingerprint and potentially improve the localization accuracy. In this paper, we exploit multiple RSSI measurements like the authors in [51], expecting to remove the noise and improve the localization accuracy. CNN are used taking into account the correlation between different RSSI measurements. We propose to split the studied environment into region “classes” limited in space, and we construct radio images from measured RSSI fingerprints. These radio images are used as our CNN model input data to predict the real-time region index. The main contributions of this paper are summarized as follows.

- An advanced cost-effective indoor localization framework inspired by the image classification process is developed using CNN for region recognition on radio tensors based on collected RSSI data.
- To the best of our knowledge, this is the first time that radio tensors (used as CNN localization system inputs) have been constructed based on RSSI data alone, without exploring extra information (magnetic information, acceleration, visual data, etc.). This avoids modifications of the existing infrastructure and the increase in the cost of the proposed solution. For this, we propose to use the kurtosis values calculated from measured RSSI. By using the kurtosis, we aim to provide a statistical parameter that will give global information to local filters. This choice is justified later, empirically. Furthermore, the proposed approach is independent of the communication technology because in all of them, the RSSI can be measured.
- To train CNN models, multiple datasets are used where each dataset corresponds to a specific training point, presenting RSSI values received from different APs during *T*. The parameter *T* is varied in order to study its impact on the localization accuracy and choose the best value considering the trade-off between localization accuracy and computational complexity. This is the first time that such an input data structure has been introduced analyzing the impact of *T* on localization performance.
- Our implemented classification network uses radio tensors to predict the index of the region containing the target. For this, our environment can be split into different grid sizes and forms, without the need to add or place APs in specific positions. Thus, we develop a flexible framework that can be applied to any existing indoor environment. As mentioned before, existing approaches based on region recognition are not flexible and require the use of extra hardware.
- Simulation results based on a realistic propagation model are presented. Different parameters are empirically justified. Finally, the localization accuracy associated with different indoor localization approaches is compared in order to illustrate the outperformance of the proposed one.

The remainder of this paper is organized as follows: In Section 2, we present the system model and explain each step of our developed framework based CNN. In Section 3, the architecture and different aspects of CNN are presented. The obtained results are presented and discussed in Section 4. Finally, the conclusion is given in Section 5.

## 2. System Model of the Proposed CNN-Based Localization Framework

**Figure 1:**
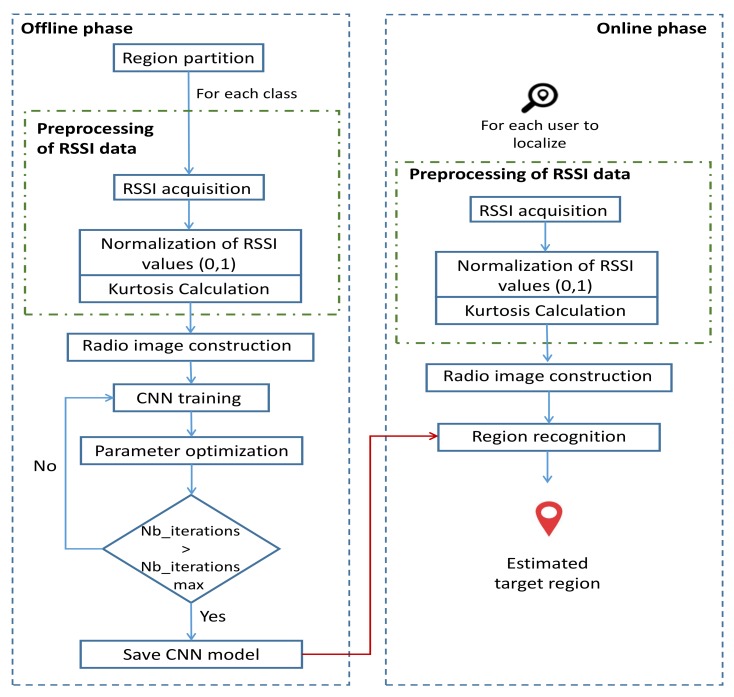
Different steps of our CNN-based localization system.

**Figure 2:**
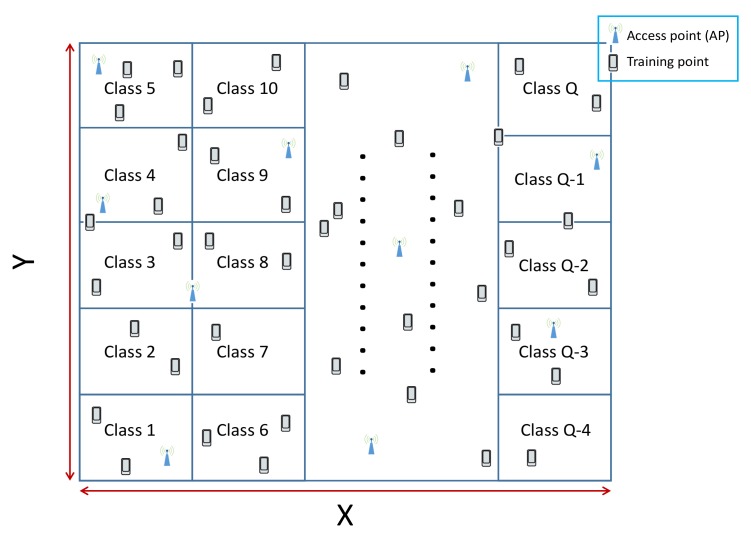
Region partition.

Our system model included two phases (Figure 1): an offline phase including the collection and preprocessing of data to be used as inputs for the localization CNN model and the training of the latter and an online phase introduced to find the position of each sensor node in the studied area using the trained model. First, the studied area was split into different partitions named “classes”, as shown in Figure 2. Each region was labeled class *q* with q∈1,2,…,Q, and *Q* is the number of classes. We mention that the area of each class is a choice, based on the precision of localization required by the application and the availability of computing resources.

RSSI values received from *M* deployed APs at different training points (sensor nodes) were measured at different instants. After acquiring RSSI measurements, a normalization process was conducted in order to make all RSSI values be included in [0,1]. RSSI matrix was used as two dimensions (2D) of the radio tensor. Then, a statistic parameter named “kurtosis” was calculated and added in the third dimension (3D) of the radio tensor, leading to the development of two localization frameworks: without and with the kurtosis plane, referred to as CNNLocWoC and CNNLocWC, respectively. After organizing the inputs of CNN, which is a crucial step, many CNN architectures were implemented and tested in order to ensure a satisfying localization accuracy. Finally, a sensor node could be localized efficiently using the considered trained model. In our system, the localization was formalized as a classification problem with *Q* classes. Each step is explained and described in detail later.

### 2.1. Preprocessing of RSSI Data

Preprocessing of RSSI data refers to transformations on the input data before they are fed to the CNN model. Different steps and techniques, applied in order to speed up training and to lead to good classification performance, are described in detail (RSSI acquisition, RSSI normalization, and kurtosis calculation).

#### 2.1.1. RSSI Acquisition and Normalization

**Figure 3:**
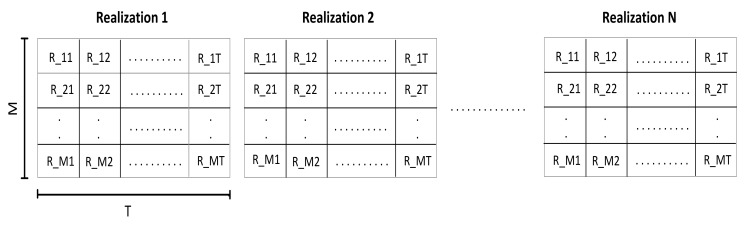
The structure of RSSI databases at each training point.

At each training point, *T* consecutive RSSI measurements, received from *M* APs, were taken. *N* RSSI databases, called realizations, were constructed for each training point, as illustrated in Figure 3; where *N* is the number of realizations, *T* is the number of RSSI measurements received from each AP, and *M* is the number of APs. Therefore, each realization presented RSSI values received from different APs at *T* instants. Notice that *N* and *T* were experimentally adjusted.

The most important factor in deep learning is how much data are available for training and how relevant they are. Furthermore, data are generally required to be normalized, especially when using gradient-based optimization methods, in order to accelerate the learning process and minimize the risk of algorithm divergence [52,53].

#### 2.1.2. Kurtosis Calculation

In order to improve the efficiency of our developed framework, we considered using the kurtosis as the third dimension of our tensor, because we wanted to introduce new information to our network. By using the latter, we aimed to provide statistical information calculated from RSSI values that can present useful information (global information of the input image). The kurtosis brings nonlinear information, which can be useful and non-redundant since the operations ensured by a neural network are linear operations. It was defined by Karl Pearson as the fourth moment [54]. $R_{mt}$ is the RSSI value received from AP *m* at instant *t*, where m=1,2,…,M and t=1,2,…,T. For a specified sensor node, the kurtosis is calculated as follows:

(1) $$kur_{mk}=\frac{1}{T}\times \sum_{t=1}^{T}{\left(\frac{R_{mt}-\mu_{k}}{\sigma_{k}}\right)}^{4},$$

where:

(2) $$\mu_{k}=\frac{\sum_{m=1}^{M}R_{mk}}{M},$$

and:

(3) $$\sigma_{k}=\frac{\sum_{m=1}^{M}R_{mk}^{2}}{M},$$

### 2.2. Radio Image Construction

**Figure 4:**
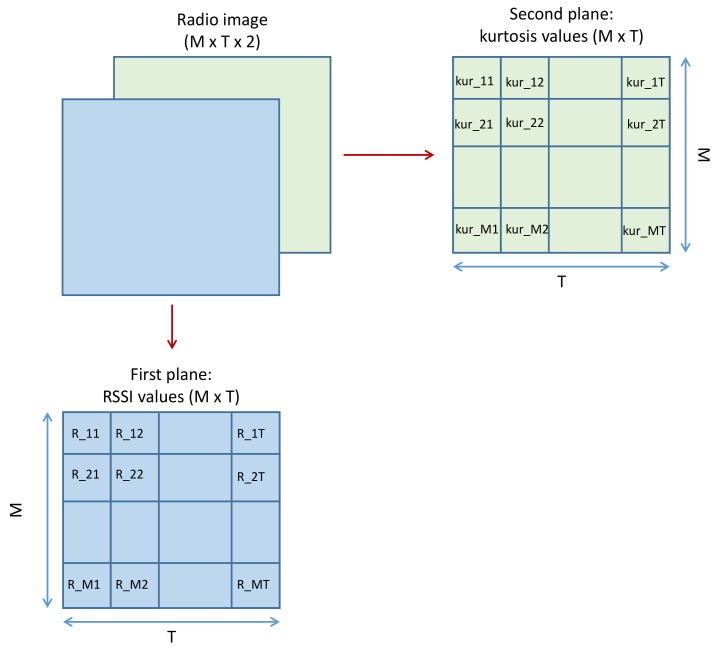
The structure of the radio images.

After collecting RSSI values and calculating the values of kurtosis corresponding to each RSSI database forming a radio tensor, 3D radio images were constructed. As the two first dimensions, we put *T* measured RSSI values from *M* APs, and we put kurtosis values in the third dimension. Thus, the size of each realization became (M×T×2) (Figure 4). Constructed radio images needed to be classified and organized, so each image was labeled *q*, q=1,2,…,Q. Then, *N* realizations of each sensor node should belong to the associated class. Images were organized into *Q* folders labeled class1,class2,…,classQ, each containing the appropriate radio images.

### 2.3. Model Training and Target Localization

Training refers to finding the best set of weights that maximize the model’s accuracy. This is related to maximizing the classification score. For this, a backpropagation process associated with an optimization algorithm was used. After training (in the offline phase), our model was able to localize a sensor node accurately in its target area. In the next section, we discuss the CNN architecture, the training process, and some design aspects.

To find a sensor’s position, after acquiring RSSI values and doing the preprocessing of the data, a radio image was constructed having the same dimension and structure of those used for training. This image was fed to the trained model in order to predict the region to which the sensor node belonged. For this, probabilities were assigned to each class, and the sum of these probabilities was equal to one. The predicted class was the one that corresponded to the highest probability.

## 3. Deep CNN Architecture Overview

**Figure 5:**
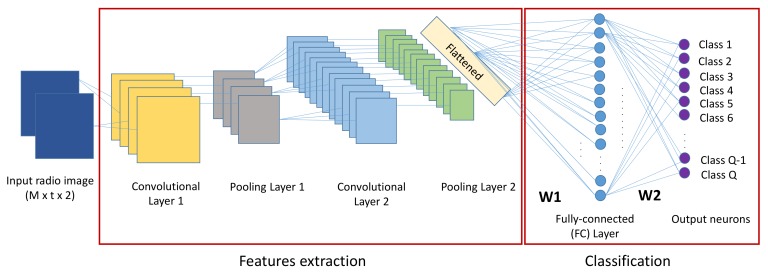
An example of a CNN architecture with two convolution layers and one fully-connected layer.

The Convolutional Neural Network (CNN) is a part of the Deep Neural Networks (DNN), including specialized NN layers, where each layer ensures a specific function. The structure of a convolutional neural network designed for region recognition consists of one or more convolution layers followed by one or more fully-connected layers taking radio images as the input and the classes’ labels as the output neurons. We explain the detailed role of each layer of the system model shown in Figure 5.

### 3.1. CNN Layers (Convolutional, Pooling, and Fully-Connected Layers)

As said before, CNN consists of multiple hidden layers between the input and output layer. The hidden layers consist of convolutional layers, pooling layers, and fully-connected layers. The role of each layer is described in detail.

#### 3.1.1. Convolution Layer

After the input layer, which takes the radio tensors, a typical CNN’s structure was designed beginning with a feature extraction process. This feature extraction, of the input tensor function, was ensured by a randomly initialized filters. Multiple filters could be used to extract the maximum of features and characteristics contained in the input data. After sliding (convolving) filters across the input’s pixels, each convolutional output was fed to an activation function. The current default choice for activation functions in CNN, namely Rectified Linear Units (ReLU), was used. It was applied to handle non-linearity in the data. It is given by:

(4) $$f_{ReLU}\left(x\right)=\left\{\begin{array}{cc} x & \;\mathrm{if}\;x>0\\ 0 & otherwise \end{array}\right.,$$

where *x* is the argument of the function (in our case, *x* denotes each pixel of the convolutional output radio images).

#### 3.1.2. Pooling Layer

**Figure 6:**
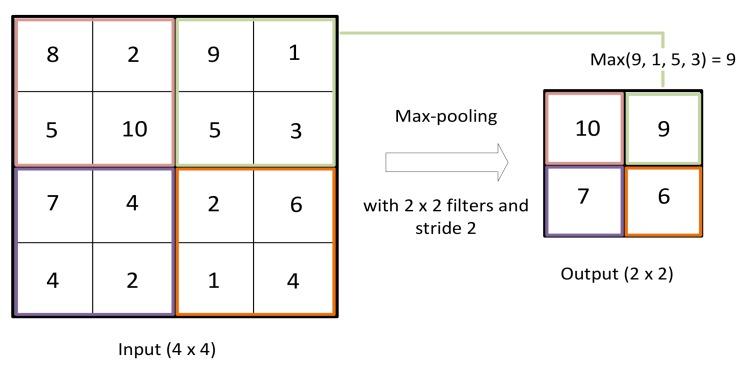
Max-pooling operation on radio images (2 × 2 window and stride two).

This layer is a spatial reduction layer that downsamples the outputs of the previous convolutional layer. It reduces the computational load and the time complexity by reducing the dimension of tensors obtained as outputs of the previous convolutional layer. We chose the max-pooling function, which selects the maximum value of the ones covered within the current pooling chosen window (Figure 6). When we use a small-sized tensor and we want to learn all the features from the entire sensor, this layer can be eliminated.

#### 3.1.3. Fully-Connected Layer

After a feature extraction process provided by a combination of convolutional layers and spatial reduction layers, a fully-connected layer was in charge of identifying the classes probability using the softmax function [55]. The class with the highest probability was selected as the output (in our case, our classes were the formed partitions). In this layer, the neurons were all connected to the neurons in the previous layer.

The number and the size of filters, the number of layers (convolutional and fully-connected layers), and various hyperparameters of CNN are adjusted by simulations and discussed in Section 4. The optimization of different parameters was a crucial step in the CNN’s training process, which is an empirical process requiring several simulations.

### 3.2. CNN Optimization

In the training phase, our CNN model used a backpropagation algorithm. The weights *w* were updated iteratively in order to reduce the loss function, between the initial prediction (estimated class) and the label (real class), most efficiently using Stochastic Gradient Descent (SGD) [56], Root Mean Squared Propagation (RMSProp) [57], and Adaptive Moment estimation (Adam) [58]. Gradient descent is the most common first order optimization algorithm in machine learning and deep learning. RMSProp and Adam are first order gradient-based optimization of stochastic objective function algorithms. They are advanced methods used to optimize the learning process registered by SGD employing an adaptive learning rate.

#### 3.2.1. Stochastic Gradient Descent

Gradient descent aims to find the local minimum of differentiable cost function *J*. GD is based on updating weights *w* in the direction to optimize the objective function J(w), using a constant learning rate for every weight update. The new parameter $w^{\left(i+1\right)}$ can be adjusted as:

(5) $$w^{\left(i+1\right)}=w^{\left(i\right)}-\alpha \nabla \left(J\left(w^{\left(i\right)}\right)\right),$$

where α is the learning rate from range (0,1), $w^{\left(i\right)}$ is the weight at iteration *i*, and $\nabla (J\left(w^{\left(i\right)}\right))$ is the gradient of the cost function with respect to the weight. Since we used the quadratic error as the cost function, $\nabla (J\left(w^{\left(i\right)}\right))$ is the difference between the estimated output $y^{\left(i\right)}$ and the wanted output $z^{\left(i\right)}$.

#### 3.2.2. Root Mean Squared Propagation

RMSProp uses recent past gradients computed in a restricted time and adjusts the weights based on how fast the gradient changes. Each weight $w_{j}$ is updated individually. For each $w_{j}$:

(6) $$w_{j}^{\left(i+1\right)}=w_{j}^{\left(i\right)}-\vartheta \times \nabla \left(J\left(w_{j}^{\left(i\right)}\right)\right),$$

where:

(7) $$\vartheta =\frac{\alpha }{\sqrt{G_{j}^{\left(i\right)}+\epsilon }},$$

where ϵ is a regularization parameter and $G_{j}^{\left(i\right)}$ is given by:

(8) $$G_{j}^{\left(i\right)}=\rho \times \sum_{j=1}^{i}{\left(d_{j}^{\left(i\right)}\right)}^{2}+\left(1-\rho \right)\times {\left(d_{j}^{\left(i\right)}\right)}^{2},$$

where ρ is a moving average adjustable parameter and $d_{j}^{\left(i\right)}$ is calculated as follows:

(9) $$d_{j}^{\left(i\right)}=\nabla \left(J\left(w_{j}^{\left(i\right)}\right)\right).$$

#### 3.2.3. Adaptive Moment Estimation

Adam uses the first gradient moment $g_{j}^{\left(i\right)}$ and the second gradient moment $v_{j}^{\left(i\right)}$ of the past gradients to adjust the weights, adding an accelerator term. The updated weight is given by:

(10) $$w_{j}^{\left(i+1\right)}=w_{j}^{\left(i\right)}-\alpha \times \frac{\frac{g_{j}^{\left(i\right)}}{1-\beta_{1}}}{\sqrt{\frac{v_{j}^{\left(i\right)}}{1-\beta_{2}}+\epsilon }},$$

where:

(11) $$g_{j}^{\left(i\right)}=\beta_{1}\times g_{j}^{\left(i-1\right)}+\left(1-\beta_{1}\right)\times d_{j}^{\left(i\right)},$$

and:

(12) $$v_{j}^{\left(i\right)}=\beta_{2}\times v_{j}^{\left(i-1\right)}+\left(1-\beta_{2}\right)\times {\left(d_{j}^{\left(i\right)}\right)}^{2},$$

We mention that $\beta_{1}$ and $\beta_{2}$ are hyperparameters of Adam, adjusted by simulations.

### 3.3. CNN Overfitting Considerations

In deep learning, overfitting is a problem encountered when our model is not able to generalize and predict the output accurately. To prevent overfitting, different interventions can be taken into account. For this:

- We introduced a dropout rate. This is ignoring some subset of neurons in a given layer in training, i.e., dropping the nodes from the layer at each training stage. In our proposed models, dropout was used after each fully-connected layer. The dropout regularization rate is mentioned in each case in Section 4.
- We added more data in the training set to be able to learn more from the training set and to add more diversity without redundancy.

## 4. Simulation Results

In this section, we present different obtained results. Furthermore, we justify empirically the choice of the different parameters used, the optimization algorithm, and the architectures.

### 4.1. Simulation Setup

We considered a wireless sensor network of *M* access points (in our simulations, we worked with five and 10) and *L* training points, placed in an area of 400 m${}^{2}$ (i.e., 20 m × 20 m). It was partitioned on grids of dimension 5 m × 5 m or 2 m × 2 m. When we worked with a grid of size 5 m × 5 m, we considered four training positions per class; while there was one training point per class when the size of the grid was 2 m × 2 m.

The training node locations and AP locations were randomly placed in the studied area. The accuracy was investigated over many environment realizations. In order to simplify the presentation of the paper and without loss of generality, we describe one environment test, as shown below, where the choice of each parameter was justified experimentally. Different RSSI measurements $R_{ml}$ received from AP *m*(m=1,2,3,…,M) were taken at each training position *l*(l=1,2,3,…,L) for sigma shadowing equal to two, using a real propagation model and conducting intensive simulations aiming to simulate real propagation conditions. The value of RSSI was calculated in dBs as:

(13) $$R_{ml}=p_{e}-{\mathrm{pl}}_{ml}+x_{\sigma },$$

where $p_{e}$ is the transmission power, $x_{\sigma }$ is a Gaussian random variable with zero mean and variance σ, which describes the random shadowing effects, and ${\mathrm{pl}}_{ml}$ is the path loss in dBs [59].

(14) $${\mathrm{pl}}_{ml}={\mathrm{pl}}_{0}+20log_{10}\left(f\right)+10\varrho log_{10}\left(\frac{d}{d_{0}}\right),$$

where ${\mathrm{pl}}_{0}$ is the path loss value at a reference distance $d_{0}$, ϱ is the path loss exponent, *f* is the used frequency in MHz, and d is the distance between the *m*th AP and the *l*th node. In this paper, we used parameters relative to our laboratory: ϱ=3.23, $p_{e}=20$ dBm, $d_{0}=1$ m, and f=2.4 GHz. The sigma of the random variable $x_{\sigma }$ took the value of two.

The dimension of each RSSI fingerprint as mentioned before was (M×T), where *T* was varied to choose the best value, as discussed later. The number of realizations per training point was N=72. Therefore, we obtained 72 tensors corresponding to each training sensor node, and the dimension of each tensor was (M×T×1) with the CNNLocWoC method and (M×T×2) when applying the CNNLocWC method. Eighty percent of tensors were considered for the training phase and 20% for validation. The presented performances were based on the validation data.

Our experiments were conducted on a PC with Intel(R) Core(TM) i7-6700 CPU @3.4 GHz. MATLAB R2018a has an advanced Neural Network Toolbox. It is a very efficient framework for us to implement our CNN models.

### 4.2. Hyperparameter Settings

The optimization of hyperparameters and the architecture choice are the most important factor in the CNN’s performance. Identifying the optimal values of the CNN parameters is defined by an empirical process. Thus, it required several experimentations. Estimated values depend on the input data. Data were trained with different numbers of convolutional and fully-connected layers to find the best architecture.

Since training the CNN model was the most time-consuming process of implementing our system, we fed mini batches of the input data, rather than exposing the entire data, to the network during consecutive learning iterations in order to accelerate training [60,61]. Therefore, an iteration corresponds to passing a mini batch of data. In this way, we ensured more robust convergence compared to the full batch learning algorithms [62]. An epoch is a full pass through the entire data. The max number of iterations to reach convergence is given by Equation (15). We chose the value of mini batch size properly because increasing the batch size decreased the time of convergence and performed better. However, from a certain point, the system can find problem of generalization. Therefore, determining the appropriate size of mini batch is a crucial step in CNN networks.

(15) $$iterations=\frac{input\;data}{mini\;batch\;size}\times epochs.$$

The number of filters, the filter size, the pooling size and stride, the mini batch size, and the number of epochs were optimized during the training phase. For each fully-connected layer, we adjusted the number of neurons and the dropout regularization rate to retain the best configuration. As the final configuration, we chose the best architecture with its appropriate parameters. The parameters illustrated in Table 1 are the same for all trained models. For the others not mentioned (the number of filters, the mini batch size, the number of epochs, the number of neurons in the fully-connected layer, and the dropout regularization rate), their values depended on the trained model.

### 4.3. Evaluation of Localization Accuracy

In the literature, machine learning-based localization approaches can solve regression or classification problems. In this paper, motivated by the outstanding performance of CNN in the image classification problem, we dealt with the issue of indoor localization as 3D radio image-fingerprint-based location recognition. Thus, as the output of our CNN model, we had the label of the predicted class containing the sensor node to localize, and not its position coordinates. To evaluate the performance of such deep learning algorithms, standard metrics were calculated [63] comparing the actual classes and the assigned ones. In this paper, we introduced accuracy as a classification performance metric. It is **the percentage of correctly-classified** sensor nodes; it refers to the recognition rate of the classifier. It is defined as:

(16) $$accuracy=\frac{C_{true}}{C_{total}}\times 100,$$

where $C_{true}$ is the number of sensor nodes rightly classified and $C_{total}$ is the total number of sensor nodes classified.

#### 4.3.1. Optimization Algorithm

We mention that α, ϵ, ρ, $\beta_{1}$, and $\beta_{2}$ are the optimization algorithms’ adjustable parameters, selected to ensure the best result in terms of localization accuracy on the validation data. Thus, several simulations were required to identify the optimal value of each parameter. The parameters used in the rest of the paper are presented in Table 2.

In the first set of simulations, we investigated the performance, in terms of localization accuracy, of different cited optimization algorithms in order to determine the best one. SGD, RMSProp, and Adam (Table 3 and Table 4) provide the good localization accuracy in the cited scenarios. As said before, RMSProp and Adam provide an adaptive learning rate aiming to optimize the learning process registered by SGD.

We notice that the performance of RMSProp was close to that obtained when using SGD. However, Adam slightly outperformed the latter. In terms of computational complexity and running time, the highest ones went to RMSProp, and this can be a major inconvenience, especially when we work with large networks and small grids, which require the use of hardware with huge computational capabilities. Adam had the lowest complexity; it accelerated the convergence compared to SGD and RMSProp. Instead of converging at 3800 iterations or 1900 iterations as SGD and RMSProp, respectively, it converged at 285 iterations when working with a grid of size 2 m × 2 m and 10 APs. Thus, Adam will be considered in the rest of this paper as the optimization algorithm.

#### 4.3.2. Fingerprint Construction

We tried to construct a significant training database, which included several RSSI variations, in order to present the maximum of fluctuations and variations, since indoor environments are characterized by high dynamics of people and other structural changes. The fingerprint database can be updated periodically depending on the availability of computing and memory resources. We studied the impact of the variation of our input data’s size. Thus, we studied the variation of parameter *T* on the localization accuracy, and we proved empirically the beneficial use of kurtosis.

##### (A) Variation of the Parameter *T*

**Figure 7:**
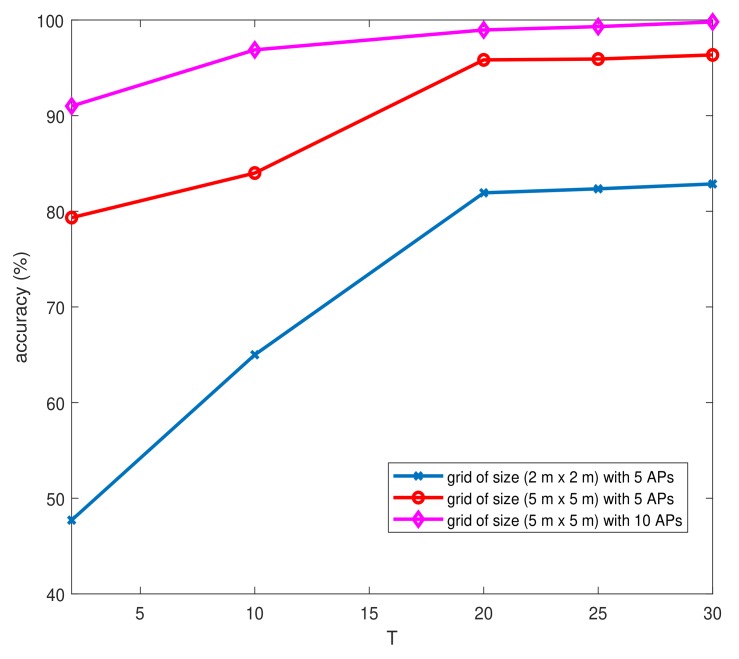
Variation of the accuracy depending on the parameter *T*.

As said before, we worked with RSSI databases of size (M×T). In this section, we study the impact of the variation of *T* on the localization accuracy taking into account the complexity and the training time. To determine the best localization accuracy and reduce the training time, we worked with four simulation scenarios: 10 APs and a grid of size 5 m × 5 m, 5 APs and a grid of size 2 m × 2 m, 5 APs and a grid of size 5 m × 5 m, and 10 APs and a grid of size 2 m × 2 m. We used a two convolutional layer architecture and one FC layer. Figure 7 illustrates the accuracy of localization related to the parameter *T*.

We notice that the localization accuracy became almost stable from T=20. We notice that the training time increased considerably from T=20 (Table 5 and Table 6). The trade-off between localization accuracy and complexity led us to use a parameter *T* equal to 20. We mention that our computing and memory resources did not allow us to construct RSSI databases with T>20 for 10 APs and a grid of size 2 m × 2 m. However, due to the fact that T=20 was proven to be the best value of parameter *T* for the three other simulation scenarios, as shown in Figure 7, we generalized this result.

##### (B) Empirical Proof of the Beneficial Use of Kurtosis

In this section, we demonstrate the advantage of the use of kurtosis on the localization accuracy, empirically. We wanted to improve the performance of Adam on the radio fingerprint, introducing a radio tensor. Table 7 illustrates the obtained performance when using the kurtosis (CNNLocWC) and without kurtosis (CNNLocWoC), on the validation data. We can easily notice that the use of this parameter guaranteed an improvement of almost 2.5% of the localization accuracy and an acceleration of the convergence of the optimization algorithm, which converged in 190 iterations instead of 285 iterations. Therefore, we found that using kurtosis was a good way to enhance the localization accuracy without the need to deploy extra infrastructure. The tests were performed for several scenarios, but only one is presented in this paper.

#### 4.3.3. CNN Architecture: Variation of the Number of Convolutional Layers

To analyze the effect of the number of layers, CNN models were performed with different numbers of convolutional layers and different numbers of neurons in each layer. The mini batch size considered was 300. We used a fully-connected layer with 120 neurons and dropout regularization with a 0.3 rate after the feature extraction module. The output layer was composed of the output neurons (100 classes because we worked with a grid of size 2 m × 2 m). The results are summarized in Table 8. Different models were trained in order to find the best number of filters in each layer and presented. For the filters, we used zero padding with stride one. We note that we mean by Conv(p,q) a convolutional layer with *p* filters with size (q×q) and by max-pooling(y,z) a max pooling layer with size (y×y) and stride *z*.

It is clear that the CNN network with two convolutional layers, associated with the best localization accuracy, outperformed the others. This is based on the fact that such a network is complex enough to extract appropriate features for region recognition. A CNN network with more than two convolutional layers is a complex model tending to cause overfitting. Therefore, deploying a CNN network with two layers seems to be the best architecture to obtain a good localization accuracy.

### 4.4. Comparison of the Indoor Localization Accuracy of Different Approaches

The proposed indoor localization method based on CNN using RSSI values needed to be evaluated and compared to standard methods. All methods used the RSSI information to localize a specific sensor node. For the trilateration technique, it was based on pairwise distances between the node to localize and APs, requiring at least three known pairwise distances. Based on traditional NN, we introduced two systems “Classic NN” and “Classic NN2”. Classic NN was a network composed of five FC layers. It was associated with the same order of complexity, in terms of the number of weights to learn, registered by CNN in order to compare these techniques fairly. Classic NN2 was verified experimentally to be the best NN model implemented based on our data. To reach this model, we began with a one-FC layer model. The best localization accuracy 83.3% was obtained with 120 neurons. Then, a two-FC layers’ model was constructed. The number of neurons was optimized to reach 84.5% accuracy. A third FC layer was added, contributing 84.75% accuracy. From four FC layers, it decreased again to reach 80%. Therefore, we worked with the three-FC layer model, which had the best accuracy. Table 9 presents the localization accuracy associated with each technique, in order to compare the performance of our developed localization frameworks (CNNLocWoC and CNNLocWC) and other existing approaches that did not use the kurtosis information (trilateration, Classic NN, and Classic NN2). The deep learning network architectures associated with the presented results are presented in Table 10.

We notice easily that trilateration introduced the worse localization accuracy compared to the other tested algorithms. Classic NN was associated with good localization accuracy, but it was less accurate than indoor localization systems based on CNN. To reach good accuracy, we had to feed the model appropriately because we could get better accuracy with lower complexity (Classic NN2 was better and less complex than Classic NN), especially since the considered radio tensors were not big. For CNN, it was associated with the best localization accuracy. When used with kurtosis, only 5.84% of classes were wrongly estimated. From each, 96.75% were estimated as neighboring class. We notice that this error of classification was always caused by training points near the class borders. Therefore, in future works, we will have to split the studied area in a way to avoid this case.

## 5. Conclusions

In this paper, a CNN indoor localization framework based on RSSI measurements was developed. We aimed to shift the online prediction complexity to an offline preprocessing step. This method investigated, not only RSSI measurements, but also the corresponding kurtosis values calculated based on RSSI, aiming to provide new information to the network. We converted radio tensors containing RSSI values, received during *T* from different deployed APs, associated with calculated kurtosis values into 3D radio images in order to realize a region recognition. The method proposed in this paper solved the problem of the high computational complexity of the traditional methods and ensured a good localization accuracy. As said before, the optimization of hyperparameters and different architectures used is the most important factor in the CNN’s performance. To identify the optimal values of different parameters, several experimentations were done. This empirical process demonstrated that a two-convolutional layer CNN model optimized by Adam was the most adapted, regarding the input data. The simulation results proved that our approach was robust and outperformed other popular methods considering the trade-off between localization accuracy and computational complexity.

New positioning opportunities can be provided based on our flexible framework. It can be introduced in order to estimate the three-dimensional orientation of the sensor node, not only the three-dimensional spatial location, in order to develop a six-dimensional positioning framework. 

## Figures and Tables

**Table 1:** List of the proposed hyperparameters.

Parameter	Value
**Number of output neurons**	16 (grid size is 5 m × 5 m) and 100 (grid size is 2 m × 2 m)
**Number of convolutional layers**	0, 2, 3, 4, 5
**Number of FC layers**	1
**Filter size**	2 × 2
**Max-pooling**	Used once after the first convolutional layer
**Max-pooling size**	2 with stride 1 or 2
**Parameter T**	2, 10, 20, 25, 30
**Optimization algorithm**	SGD, RMSProp, and Adam
**Activation function**	ReLU for convolutional layers and softmax for FC layer

**Table 2:** Optimization algorithms’ adjusted values.

Parameter	Value
**α**	0.0005
**ϵ**	$10^{-7}$
**ρ**	0.99
**$\beta_{1}$**	0.9
**$\beta_{2}$**	0.8

**Table 3:** Variation of the accuracy according to the optimization algorithm on the validation data using a grid of size 5 m × 5 m.

Number of APs	Mini Batch Size	Algorithm	Accuracy (%)	Number of Iterations
		SGD	89.93	690
**5**	60	RMSProp	90.8	1610
		Adam	**93.92**	**228**
		SGD	97.57	575
**10**	60	RMSProp	98.9	1610
		Adam	**98.96**	**228**

**Table 4:** Variation of the accuracy according to the optimization algorithm on the validation data using a grid of size 2 m × 2 m.

Number of APs	Mini Batch Size	Algorithm	Accuracy (%)	Number of Iterations
		SGD	80.43	4350
**5**	200	RMSProp	80.53	2900
		Adam	**81.93**	**435**
		SGD	91.14	3800
**10**	300	RMSProp	90.71	1900
		Adam	**91.57**	**285**

**Table 5:** Variation of the accuracy according to *T* using a grid of size 5 m × 5 m.

Number of APs	Mini Batch Size	T	Accuracy (%)	Training Time (min)	Prediction Time (s)
		2	79.34	0:06	$0.24\times 10^{-3}$
		10	84	0:07	$0.29\times 10^{-3}$
**5**	60	20	**95.83**	**0:10**	**$0.36\times 10^{-3}$**
		25	95.92	0:50	$0.83\times 10^{-3}$
		30	96.35	1:10	$0.91\times 10^{-3}$
		2	91.57	0:09	$0.3\times 10^{-3}$
		10	96.88	0:09	$0.48\times 10^{-3}$
**10**	60	20	**98.96**	**0:15**	**$0.58\times 10^{-3}$**
		25	99.31	1:23	$1.3\times 10^{-3}$
		30	99.88	1:50	$1.5\times 10^{-3}$

**Table 6:** Variation of the accuracy according to *T* using a grid of size 2 m × 2 m.

Number of APs	Mini Batch Size	T	Accuracy (%)	Training Time (min)	Prediction Time (s)
		2	47.71	0:13	$0.17\times 10^{-3}$
		10	65	0:24	$0.27\times 10^{-3}$
**5**	200	20	**81.93**	**1:25**	**$0.3\times 10^{-3}$**
		25	82.35	4:31	$0.69\times 10^{-3}$
		30	82.86	5:15	$0.93\times 10^{-3}$
		2	72.07	0:47	$0.2\times 10^{-3}$
		10	85.36	1:15	$0.28\times 10^{-3}$
**10**	300	20	**91.57**	**2:52**	**$0.68\times 10^{-3}$**
		25	X	–	–
		30	X	–	–

**Table 7:** Adam’s accuracy on the validation data using a grid of size 2 m × 2 m and 10 anchors.

	Mini Batch Size	Accuracy (%)	Number of Iterations
CNNLocWoC	300	91.57	285
CNNLocWC	400	94.13	190

**Table 8:** Variation of the accuracy according to the number of layers using a grid of size 2 m × 2 m and 10 anchors

Number of Convolutional Layers	Accuracy (%)	Feature Extraction Module Architecture
0	83.3	–
		Conv(200,2)
2	91.57	Max-pooling(2,2)
		Conv(120,2)
		Conv(120,2)
3	88.43	Max-pooling(2,2)
		Conv(200,2)
		Conv(300,2)
		Conv(40,2)
		Max-pooling(2,2)
4	83.29	Conv(90,2)
		Conv(300,2)
		Conv(400,2)
		Conv(40,2)
		Max-pooling(2,2)
5	82	Conv(90,2)
		Conv(300,2)
		Conv(400,2)
		Conv(700,2)

**Table 9:** Comparison of the accuracy associated with different algorithms using a grid of size 2 m × 2 m and 10 anchors.

Indoor Localization Technique	Accuracy (%)
Trilateration	30
Classic NN	80.76
Classic NN2	84.75
CNNLocWoC	91.57
CNNLocWC	94.13

**Table 10:** The deep learning network architectures used.

Deep Learning Algorithm	Network Architecture
	FC(1500)
	FC(3000)
Classic NN	FC(2000)
	FC(1200)
	FC(120)
	FC(100)
Classic NN2	FC(200)
	FC(120)
	Conv(200,2)
CNNLocWoC	Max-pooling(2,2)
	Conv(120,2)
	FC(120)
	Conv(200,2)
CNNLocWC	Max-pooling(2,2)
	Conv(300,2)
	FC(120)
